# Neutrophil unsaturated fatty acid release by GM-CSF is impaired in cystic fibrosis

**DOI:** 10.1186/1476-511X-9-129

**Published:** 2010-11-08

**Authors:** Elena Bravo, Mariarosaria Napolitano, Sara Benedetti Valentini, Serena Quattrucci

**Affiliations:** 1 Department of Cell Biology and Neurosciences, Istituto Superiore di Sanità, Viale Regina Elena, 199 - 00161 Roma, Italy; 2 Centro Fibrosi Cistica di Riferimento Regione Lazio - Department of Pediatrics -University "La Sapienza", Viale Regina Elena 324 - 00161 Roma, Italy

## Abstract

Dysregulated inflammation in cystic fibrosis (CF) is attributed to an altered production of inflammatory mediators derived from polyunsaturated lipids. In comparison to the arachidonic acid (AA) cascade, little is known about the modulation of docosahexaenoic acid (DHA) membrane release. We compared data on neutrophil DHA- and AA- release from both control (CT) and patients with CF using [^3^H]AA or [^14^C]DHA as a markers for, respectively, AA and DHA- release. Granulocyte-macrophage-colony stimulating factor stimulated DHA release from CT, but not CF, neutrophils. Comparison showed that both [^14^C]DHA and [^3^H]AA liberated after stimulation was higher in CT than in CF neutrophils. Since bioactive mediators derived from DHA are resolving factors and those derived from AA are both pro- and anti- inflammatory, these results suggest that CF is associated with a reduction of the release of PUFA-precursors of lipooxygenated resolving mediators. This leads to the hypothesis that defects in the resolving factors production could contribute to the inflammatory dysregulated processes in CF. Furthermore, the methodology used may help to improve knowledge on the regulation and resolution of inflammation.

## Introduction

Generation of inflammatory mediators is regulated by cytokines and factors released in the early phase of inflammatory and infectious processes. The precursors of bioactive lipid mediators are the polyunsaturated fatty acids (PUFA) released from membrane phospholipids. Earlier studies of bioactive lipids introduced the concept that arachidonic acid [20:4, n6; AA] released from membrane phospholipids by cytosolic phospholipase A2 (cPLA2) is transformed to several series of potent bioactive eicosanoids: prostaglandins, leukotrienes and lipoxins [1]. In more recent years, novel enzymatic oxygenated products generated in vivo were identified in pathways initiated from the precursors eicosapentaenoic acid and docosahexaenoic acid (C22:6 n3; DHA). These new families of compounds contribute functionally to the resolution of inflammation [2-4]. As the discovery of these molecules is relatively recent and the direct analyses of these compounds requires Liquid Chromatography with Tandem Mass Spectrometry Detection [3], most of the available studies have been focused the regulation of AA cascade, while the modulation of DHA metabolism has been poorly investigated and, as a consequence, the mechanisms modulating DHA release are largely unknown.

Knowledge of these mechanisms could be particularly important for Cystic Fibrosis (CF) patients. CF is the most common lethal autosomal recessive hereditary disease in Caucasians and it is due to dysfunction of the cystic fibrosis transmembrane conductance regulator gene. The basic defect results in secondary pulmonary infection and an excessive neutrophil-driven inflammatory response which are responsible for most of the morbidity and mortality in these patients [5]. The genetic defect lead also to pancreatic insufficiency, and CF-related gastrointestinal manifestations [6].

CF is associated with an imbalance in the ratio of essential PUFA due to a decreased levels of linoleic and docosahexaenoic acid (n3C22:6; DHA) and to a variable excess of arachidonic acid (n6 C20:4; AA) with respect to DHA [7].

Since in a previous study we showed in CF a lower neutrophil AA-generation in response to lipopolysaccharide (LPS) [8], in this study we aimed to investigate the modulation of DHA release in CF neuttrophils using a similar approach to that used to elucidate the regulation of AA metabolism, and in particular:

a. to study the release of DHA and AA from neutrophils in response to granulocyte-macrophage-colony stimulating factor (GM-CSF) challenge;

b. to compare the DHA and AA release between neutrophils isolated from control subjects (CT) and CF patients.

## Materials and methods

Arachidonic acid-5,6,8,9,11,12,14,15-^3^H(n) (180-240Ci/mmol), 4,7,10,13,16,19-Docosahexaenoic acid-1-^14^C (40-60 mCi/mmol), and various classes of products were purchased from Sigma Chemical Company (St. Louis, MO, USA). Medium and supplementing factors were obtained from Hyclone Europe Ltd (Sial, Rome, Italy). Dextran T-70 was obtained from Pharmacia Pfizer Italy. GM-CSF was purchased from R&D Systems Europe Ltd (Space Import Export S.r.l, Milan, Italy).

The study was approved by the Ethical Committee of the University La Sapienza. Written consent was obtained by patients and controls. 15 CF patients and 18 CT, with an average age of 27 ± 7 and 28 ± 7 years, respectively, were enrolled in the study. Only non smokers, or subjects that had not smoked for at least 1 year, were admitted to the study. Diagnosis of CF was confirmed by at least two abnormal sweat tests. The CF genotype and general characteristics of the patients are reported in Table 1. All patients were in a stable medical condition, under standard CF therapies such as pancreatic enzyme and vitamin supplementation and did not have acute infection (negative C-reactive protein). None of them were taking corticosteroids therapy. None of CT subjects were on pharmacologic therapies and/or dietary polyunsaturated fatty acids (PUFA) supplementation.

**Table 1:** Cystic fibrosis patient characterisation

Patient	Age	Sex	Genotype	BMI^a ^(Kg/m)	Diabetes	FEV1^b ^(Lt)
1	20	F	DF508/852del22	20,4	NO	2,19 (75%)

2	40	M	G542X/UN	20,7	NO	0,49 (14%)

3	33	F	DF508/1717-1GToA	21,0	YES	2,68 (98%)

4	25	F	G542X/N1303K	17,0	YES	0,62 (22%)

5	22	M	DF508/L732X	20,0	YES	1,23 (31%)

6	34	M	UN/UN	18,0	YES	0,63 (15%)

7	27	F	N1303K/N1303K	15,0	YES	0,65 (20%)

8	33	F	N1303K/UN	20,0	YES	0,53 (20%)

9	26	F	DF508/DF508	19,0	YES	1,04 (35%)

10	36	M	G551D/621+1GtoT	21,6	NO	1,61 (37%)

11	21	F	DF508/DF508	16,3	NO	1,35 (38%)

12	15	F	DF508/UN	18,3	NO	1,05 (38%)

13	27	F	DF508/DF508	22,5	NO	1,39 (45%)

14	21	F	DF508/DF508	19,4	NO	0,80 (27%)

15	36	M	DF508/G542X	21,9	YES	1,35 (46%)

^a ^BMI: Body Mass Index; ^b ^Forced Expiratory Volume in 1 Second

Neutrophils were isolated from blood samples collected in EDTA according to Boyum [9]. After sedimentation in 3% dextran, the leukocyte-enriched pellet was layered over a Histopaque 1.077 g/ml and centrifuged at 400 × *g *for 40 min. Residual erythrocytes were lysed. The purity of CD16+ neutrophils (phycoerythrin conjugated CD16 and isotype-matched Ig from Becton Dickinson, Milan, Italy) was greater than 90%.

The first step in the generation of biologically important eicosanoids in human neutrophils is AA release from membrane phospholipids by cytosolic phospholipase A2 (cPLA2) [10]. Since AA competes with DHA for the same enzymes and for the site of esterification at the sn-2 position of phospholipids, to study [^14^C]DHA and [^3^H]AA-release, we adapted the method reported by DiPersio et al. [9]. Neutrophils PUFA release was stimulated with GM-CSF at the concentration of 0.2 nM. These conditions have been shown to be a suitable stimulation for these tests [11]. Neutrophils, at a density of 3×10^6 ^cells/ml in RPMI containing 0.3% bovine serum albumin (BSA), were radiolabelled with [^14^C]DHA (0.4 μCi/ml) or [^3^H]-AA (2 μCi/ml) for 3 h at 23°C. After labelling and washing, the [^14^C]DHA or [^3^H]AA release was tested in RPMI 0.3% BSA in resting (NT) cells or in cells challenged with 0.2 nM GM-CSF. After 30 min, duplicate samples of 200 μl centrifuged medium were assayed for radioactivity by liquid scintillation counting. Radioactivity associated with neutrophils was counted after cell lysis with 200 μl of distilled water. Extracellular [^14^C]DHA and [^3^H]AA-release is expressed as a percentage of the total radioactivity incorporated by the cells. Total radioactivity incorporated into neutrophils takes account of the activity released into the medium. Each experimental point was performed in duplicate.

Plasma fatty acid analysis was performed by gas chromatography [12]. After lipid extraction and acid catalyzed transesterification, plasma fatty acid methyl esters were analyzed by gas liquid chromatography in a Hewlett-Packard 5890 Series II gas chromatograph equipped with a flame ionization response detector, a capillary column OmegawaxiM 320 (30 m×0.32 mm) and a 0.25 mm film (Supelco, Inc.). Heptadecanoic acid (C17:0) was used as internal standard.

Values are expressed in means ± SD. Statistical analysis and correlation analysis was performed with GraphPad Instat Ver. 4.03 software. The within group differences were assessed by a paired sample paradigm by Wilcoxon matched pairs test. The comparison between the two independent groups CT and CF data was performed either by nonparametric one-way ANOVA (Kruskal-Wallis Test) using Dunn's Multiple Comparisons test as post-test or by unpaired t test with Welch correction. Pearson product moment correlation coefficient was used to evaluate the correlation between different variables.

## Results

Gas-chromatographic analysis showed that the percentage of AA (n = 15) and DHA (n = 18) relative to the total fatty acid content of plasma were, respectively, 7.12 ± 1.39% and 1.67 ± 0.81% in CF patients and 6.72 ± 2.28% and 1.76 ± 0.79% in the CT group, without differences between the groups. However, as expected in these patients, the plasma AA/DHA molar ratio (4.87 ± 2.42 and 3.60 ± 0.81 in CF and CT group, respectively) was significantly higher in CF (P < 0.05) with respect to CT.

The [^14^C]DHA release (Table 2) from the membrane by resting CF neutrophils was not significantly different than CT cells. However, [^14^C]DHA release was increased upon stimulation with GM-CSF in CT neutrophils, but was scarcely modulated in the CF group. The differences, in fact, between [^14^C]DHA release in NT and GM-CSF conditions were statistically different in the CT group, but not in the CF group (P < 0.002 and P = 0.09 in CT and CF, respectively) and consequently, release was significantly higher in CT as compared to CF cells after GM-CSF stimulation (Table 2). In both CF and CT groups, [^14^C]DHA release by resting neutrophils was not correlated with the release upon GM-CSF stimulation, or with the proportion of DHA or AA in the plasma.

**Table 2:** Release of DHA by neutrophils

*Neutrophils [^14^C]DHA release (%)* *Mean ± SD (n)*		
	NT	GM-CSF
**CT**	11.1 ± 6.3 *(18)^a^*	15.1 ± 6.9 *(18)^b^*
**CF**	9.9 ± 8.1 *(15)*	8.3 ± 4.4 *(15)*

^a ^CT: NT vs GM-CSF, p < 0.002

^b ^GM-CSF: CT vs CF, p < 0.05.

The release of [^14^C]DHA into the medium by neutrophils isolated from control subjects (CT) and patients with cystic fibrosis (CF) was measured in resting cells (NT) and after stimulation with GM-CSF (0.2 nM). Data are expressed as a percentage of the total radioactivity incorporated into the cells.

In CT neutrophils, cytokine stimulation induced a strong increase of the release of [^3^H]AA (Table 3) with respect to resting cells (NT vs GM-CSF: P < 0.001), but CF neutrophils were not responsive to the stimulation as there was no significant change in [^3^H]AA release. In fact, the mean of the [^3^H]AA released after GM-CSF challenging from CF neutrophils was markedly lower in comparison to that from CT cells (GM-CSF: CF vs CT: P < 0.02).There were no differences in either [^14^C]DHA or [^3^H]AA release between CF patient with (n = 8) and without (n = 7) diabetes.

**Table 3:** Release of AA by neutrophils

*Neutrophils [^3^H]AA release (%)* *Mean ± SD (n)*		
	NT	GM-CSF
**CT ***^a^*	6.3 ± 4.8 *(16)*	10.8 ± 5.7 *(16)^b^*
**CF**	4.1 ± 3.3 *(15)*	5.4 ± 2.0 *(15)*

^a ^CT: NT vs GM-CSF, p < 0.01

^b ^GM-CSF: CT vs CF, p < 0.02.

The release of [^3^H]AA into the medium by neutrophils isolated from control subjects (CT) and patients with cystic fibrosis (CF) was measured in resting cells (NT) and after stimulation with GM-CSF (0.2 nM). Data are expressed as a percentage of the total radioactivity incorporated into the cells.

There was a positive correlation between the [^3^H]AA released in resting conditions and after GM-CSF challenge, and this was markedly stronger in the CT group (r = 0.86, p < 0.0005) in comparison to the CF patients (r = 0.59, p < 0.05). Also, in the control group, but not the CF group, the neutrophil [^3^H]AA release positively correlated with the AA plasma content both in resting (r = 0.61, p < 0.02) and stimulated (r = 0.59, p < 0.05) neutrophils.

Evaluation of the relationship between the radioactive tests showed that [^14^C]DHA and the [^3^H]AA release were correlated, in resting condition in CF neutrophils (r = 0.80, p < 0.003), and after the stimulation with GM-CSF in CT cells (r = 0.77, P < 0.03).

## Discussion

AA and the mobilisation of its early metabolites can be monitored by the release of previously radiolabelled AA in the membrane pool. This method, described more than 20 years ago for the measurement of the pro-inflammatory precursors release by neutrophil [11,13,14], is still widely used in several fields to test membrane mobilisation of the mediator precursors [15-17], but, to the author's knowledge, it has never been used to study modulation of the DHA release. In this work we used [^3^H]AA or [^14^C]DHA as a marker for, respectively, the AA and the DHA-related products released from the membrane after neutrophil activation.

GM-CSF was found to elicit a significant enhancement of [^3^H]AA released from CT, but not CF neutrophils (Table 3). In CT neutrophils, the AA cascade triggered by GM-CSF produced a release of [^3^H]AA significantly higher than that observed in resting cells (+71%), and also than that found in GM-CSF-treated CF neutrophils (+100%). In contrast, there was no significant increase in AA-derived precursors released by the CF cells in response to specific neutrophil stimulation.

In the case of DHA, our results show that regulation of its liberation from neutrophils is also modulated in CT subjects differently than in CF patients. Treatment with GM-CSF induced a significant increase of [^14^C]DHA liberated from CT neutrophils (Table 2). Conversely, cells derived from CF patients responded to GM-CSF challenge with a slight reduction of [^14^C]DHA recovered into the medium. As mediators derived from DHA cascade induce the production of resolvins and protectins [2-4], families of compounds with resolving and anti-inflammatory activity, the lack of response to GM-CSF in CF patients suggests that the liberation of PUFA precursors of resolving mediators derived from DHA is markedly impaired in CF neutrophils.

The concept of defective production of mediator precursors in CF is supported by correlation analysis, as the releases of the [^14^C]DHA and the [^3^H]AA correlate in CF group in resting condition, whereas in CT group the same correlation is observed only after stimulation with GM-CSF, indicating that both the basal response and the mechanisms regulating precursors liberation are altered in the CF patients. Furthermore, both in resting and stimulated CT neutrophils, the [^3^H]AA released is correlated with plasma AA content, while this relationship is totally lacking in CF neutrophils, suggesting that other factors play a greater role than lack/availability of AA in the regulation of the inflammatory response in CF neutrophils. In addition, although not definitive, our results suggest that, in contrast to [^3^H]AA release, [^14^C]DHA release is not correlated with the plasma proportion of either AA or DHA, suggesting that it may be difficult to regulate resolvins and protectins synthesis by modifications of availability of DHA in CF as well control subjects.

In agreement with the data that CF-related diabetes represents the most common co-morbidity in this disease [18], about 50% of CF subject were diabetics. Diabetes in CF patients depends on the basic genetic defect and shows important differences with respect to both type 1 and type 2 diabetes [18]. We found that, although disturbances in AA cascade are common in type 2 diabetes [19], there were no differences in PUFA membrane release in CF-related diabetes.

The dysregulated inflammatory response in CF is commonly considered excessive [5], and the abnormalities in AA release in CF patients have been attributed to an exaggerated production of proinflammatory mediators such as leukotrienes and prostaglandins, which exacerbated the lung infection in these patients [5,13,14]. However, in 2004 Karp et al. [20] reported an important defect in lipoxin and anti-inflammatory activity in CF airways, and suggested that in CF a deficiency of resolving factors prevents resolution of lung inflammation and impedes healing. Our results indicate that CF is associated with both an impaired regulation by GM-CSF of the release of PUFA from the membrane and decreased liberation of DHA and AA precursors, suggesting that the generation of mediators derived from DHA and AA in CF is insufficient, rather than excessive. In particular, as lipooxygenated products derived from DHA have a major function in infection termination and healing, the lack of response to GM-CSF may be directly related to an impaired resolution of infections [21]. Furthermore, the DHA deficiency associated with CF [7] may worsen the liberation of appropriate amount of DHA-derived healing factors.

In conclusion, although future studies are necessary to clarify the underlying mechanisms of altered PUFA delivery from CF neutrophils, the preliminary results of this study indicate a major defect in DHA delivery by neutrophils delivery in CF and suggests a useful methodological approach for the study of DHA metabolism, which may help to monitor the early stages of resolving factor production in response to different therapeutic interventions.

## List of abbreviations

CF: cystic fibrosis; AA: arachidonic acid; DHA: docosahexaenoic acid; GM-CSF: Granulocyte-macrophage-colony stimulating factor; PUFA: polyunsaturated fatty acids;

## Competing interests

The authors declare that they have no competing interests.

## Authors' contributions

EB and SQ designed the study, MN carried out the experiments and analysis of the data, SQ and SBV responsible for any aspect related to the patients, EB drafted the paper. All the authors contributed to the interpretation and discussion of the results related to their part of the work and critically revised the paper.
